# Red Fruits Composition and Their Health Benefits—A Review

**DOI:** 10.3390/foods11050644

**Published:** 2022-02-23

**Authors:** Fernanda Cosme, Teresa Pinto, Alfredo Aires, Maria Cristina Morais, Eunice Bacelar, Rosário Anjos, Jorge Ferreira-Cardoso, Ivo Oliveira, Alice Vilela, Berta Gonçalves

**Affiliations:** 1 CQ-VR, Chemistry Research Centre-Vila Real, University of Trás-os-Montes e Alto Douro, Quinta de Prados, 5000-801 Vila Real, Portugal; avimoura@utad.pt; 2 CITAB, Centre for the Research and Technology of Agro-Environmental and Biological Sciences, Inov4Agro, Institute for Innovation, Capacity Building and Sustainability of Agri-Food Production, University of Trás-os-Montes e Alto Douro, Quinta de Prados, 5000-801 Vila Real, Portugal; tpinto@utad.pt (T.P.); alfredoa@utad.pt (A.A.); mariacristina.morais@gmail.com (M.C.M.); areale@utad.pt (E.B.); ranjos@utad.pt (R.A.); jventura@utad.pt (J.F.-C.); ivo.vaz.oliveira@utad.pt (I.O.); bertag@utad.pt (B.G.)

**Keywords:** antioxidant activity, phenolic compounds, volatile compounds, vitamins, minerals, fatty acids, fibers, consumer perception, health benefits

## Abstract

The probability that fruit ingestion may protect human health is an intriguing vision and has been studied around the world. Therefore, fruits are universally promoted as healthy. Over the past few decades, the number of studies proposing a relationship between fruit intake and reduced risk of major chronic diseases has continued to grow. Fruits supply dietary fiber, and fiber intake is linked to a lower incidence of cardiovascular disease and obesity. Fruits also supply vitamins and minerals to the diet and are sources of phytochemicals that function as phytoestrogens, antioxidant and anti-inflammatory agents, and other protective mechanisms. So, this review aims to summarize recent knowledge and describe the most recent research regarding the health benefits of some selected red fruits.

## 1. Introduction

Nowadays, people are concerned about having a varied and healthy diet. Therefore, the acceptability and consumption of red fruits, especially the species of several families, such as Rosaceae (strawberry, raspberry, blackberry, and sweet cherry), and Ericaceae (blueberry, cranberry) has undergone significant increases not only due to their high nutritive value, characteristic taste, flavor and nutraceutical characteristics, but also due to their known health-promoting properties (as dietary sources of bioactive compounds) [1,2,3,4,5]. There is the recognition that red fruits are a good source of many bioactive ingredients and nutrients, including vitamins (vitamins A, C, and E), minerals (calcium, phosphorus, iron, magnesium, potassium, sodium, manganese, and copper), dietary fiber, and antioxidants [1,3]. They are also a rich source of bioactive compounds, and studies have shown that they have essential positive effects on the human diet and health, which could be mainly ascribed to the presence of several health-related compounds such as organic acids, phenolics, and sugars (glucose, fructose) [6,7,8]. Intake of fresh fruits enhanced both mental and physical health and facilitated the prevention of various non-communicable diseases as well, such as neurological disease, cardiovascular disease, diabetes mellitus, obesity, osteoarthritis, and some cancers [8]. For example, Huang et al. [9] showed that strawberries are a rich source of anti-inflammatory polyphenols such as anthocyanins, which have been shown to decrease the postprandial meal-induced increases in inflammation and oxidative stress in overweight healthy adults, mainly if the strawberry drink was consumed before the meal. In addition, the ease of transport due to their size makes them even more recommendable to be consumed in all situations. The qualitative and quantitative composition, the nutritional value, and consumer’s acceptability of the fruits in general and the red fruits, in particular, will vary with the species, cultivar, genotype, maturity stage, agricultural practice, environmental conditions, plant nutrition, soil conditions as well as subsequent storage conditions [3,4,5,6,7,8,9,10,11,12,13,14,15,16]. This review aims to summarize and update knowledge concerning the composition of red fruits and to describe the most recent research regarding the health benefits of some selected red fruits.

## 2. Red Fruits Composition

### 2.1. Vitamins and Minerals

Red fruit berries are the best dietary sources of bioactive compounds, namely vitamins and minerals with antioxidant properties [1,2]. Since red fruits do not usually undergo any processing to be consumed, their antioxidant properties are not reduced. According to Nile and Park [17], 100 g of the edible portion of raspberries, blackberries, or blueberries could provide more than 50% of Recommended Dietary Allowance for manganese, vitamin C (ascorbic acid), and vitamin B9 (folic acid).

#### 2.1.1. Vitamins

Vitamins are organic substances with strong antioxidant potential that our bodies cannot synthesize in sufficient quantities yet are essential for their good development, even in trace amounts. According to Rodriguez-Amaya [18], there are 14 known vitamins grouped into two large groups of molecules, fat-soluble (A, D, E, and K) and water-soluble (B group and C).

**Figure 1:**
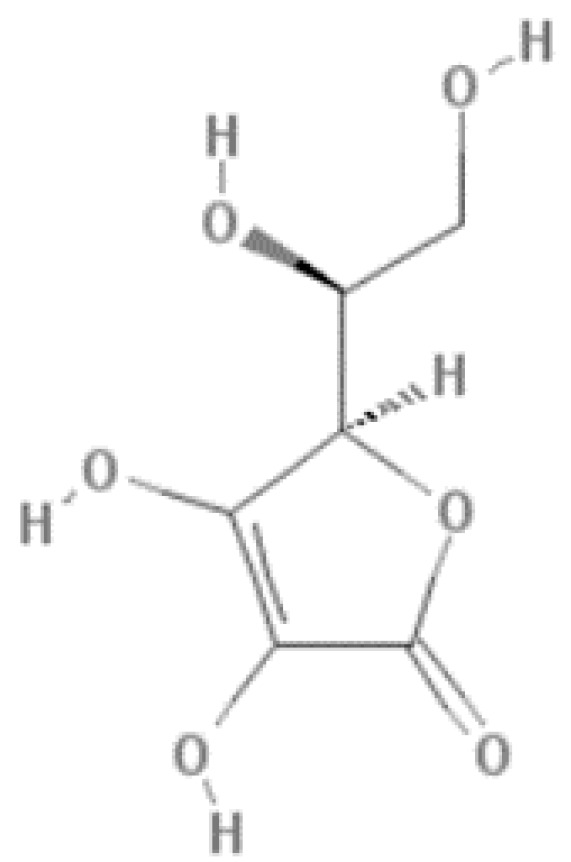
Structure of ascorbic acid.

Under the name of red fruits is a set of fruits of black or red color, mostly arranged in berries, like strawberry, cherry, red raspberry, black raspberry, blackberry, cranberry, blue-berry, blackcurrants, and grapes. Vitamin C or ascorbic acid is the most quantified in red fruits and is one of the main antioxidant compounds present in this type of fruit. It is a water-soluble carbohydrate-derived compound, known for its high antioxidant activity due to the neutralization of free radicals and other reactive oxygen species, and acidic properties due to the presence of a 2,3-enediol moiety (Figure 1) [3].

Contrary to vitamin C, vitamin A is not found in fruits, at least in large quantity, with some exceptions such as the mango, the cantaloupe, and even the watermelon. A total of 1–2 mg per day is the human requirement for vitamin B2 (riboflavin). The largest source of this vitamin is the green vegetable, unlike fruits that are relatively poor in riboflavin [3] Vitamin B6 (riboflavin) is not present in large quantities in red fruits, being present in appreciable quantities in grapes, prunes, avocados, and bananas [3].

Table 1 presents the composition in vitamins of some red fruits found by several authors, and wide variations in the contents of vitamins can be observed. For instance, the level of vitamin C ranged from 5 to 100 g/100 g fresh weight (FW) among cultivars of cherry, cranberry, blackberry, blueberry, red raspberry, and strawberry. Blueberries are the richest fruits in ascorbic acid, while the content of raspberries is similar to that of strawberries, and blackberries have a lower value of about 34 mg/100 g FW [19]. However, the ascorbic acid content in blackberry is about 2–3 times the content in red currants and about 8–9 times less than in blackcurrants [20]. Cranberry is rich in vitamins, such as vitamin C. As with strawberries, and depending on the storage conditions, cranberries also have significant losses of vitamin C after storage [21]. Cranberry is a fruit that has lately been recognized as new functional food and nutraceutical. For instance, cranberries are known to have a unique function for maintaining urinary tract health [22]. According to Dorofejeva et al. [23], in cultivated cranberries, this bioactive compound is about 10 mg/100 g dry weight (DW).

#### 2.1.2. Minerals

Usually, fruits are not recognized as primary sources of mineral intake. Even so, according to the Dietary Approaches to Stop Hypertension (DASH) fruits contribute an average of 5.8%, 17.3%, 33.0%, and 6.6% to the intakes of calcium, magnesium, potassium, and zinc, respectively [29]. Red fruits are not only a source of vitamins [1], but are also a rich source of minerals (phosphorus, calcium, iron, potassium, magnesium, manganese, sodium, and copper) [2]. Table 2 shows the mineral composition of sweet cherry, blackberry, blueberry, raspberry, and strawberry.

Among the fruits analyzed, blackberries and cherry are the fruits with the highest concentration of minerals. There are important differences between the different researchers regarding the concentration of minerals [2,19,23,30]. This is explained, as previously mentioned, since in most cases comparisons are made between fruits of different cultivars, subject to different soil and climatic conditions, and post-harvest handling techniques [2]. The effects of cultivars and cultivation conditions on the composition of strawberries are demonstrated by Hakala et al. [31].

### 2.2. Sugars and Organic Acids

Generally, fructose and glucose are the main sugars present in red fruits, while citric and malic acids are the main organic acids present in this type of fruit. Mikulic-Petkovsek et al. [32] studied the sugars and organic acids content in fruit of 25 wild and cultivated berry species and found that glucose and fructose were the most abundant sugars in berry fruits and the main organic acids were citric and malic acid. Instead, Viljakainen et al. [33] also showed significant variations in sugars and organic acids between different berry cultivars.

Table 3 presents the composition of some of the most common red fruits in sugars and organic acids addressed by different authors and it is consensual that different factors such as genotype, cultural practices, climate conditions, altitude, and season can affect the variation and content of such compounds. The data present in Table 3 and from other studies showed the occurrence and the variation in different sugars and organic acids. Glucose was the main sugar found in the strawberry, up to 80% [34], and citric acid the main organic acid in strawberry and blueberry juice, contributing with 62–84% and 73–90% of the total acid content, respectively [34,35]. In blueberries, a high content of fructose and citric acid as a main sugar and acid were also found, respectively [36]. In addition, Zhang et al. [36] also found high content of citric acid (1.862–13.424 mg/g) and quinic acid (0.147 to 5.445 mg/g) as the major organic acids present in blueberry fruits.

In cherries, the most abundant soluble sugars observed were glucose and fructose, together with the sugar alcohol sorbitol, while malic, oxalic, and shikimic acids were the most abundant organic acids [37].

In strawberry, Urün et al. [38] found high content of citric (522.4–711.5 mg/g FW) and malic acid (159.8–266.7 mg/g FW) and fructose was the sugar present in the highest concentration (2.17–4.43% of total sugars). Also, Morais et al. [39] showed enhancement in the total soluble solids content in strawberry plants inoculated with the PGPB strain Pedobacter sp. CC1.

In white and red grapes, tartaric, malic, and citric acids are the most preponderant organic acids. In a study of 24 red grape varieties [40], the titratable acidity, expressed as equivalent of tartaric acid, varied from 3.9 (‘Moreto Boal’) to 13.5 g/L (‘Tinta Miúda’ and ‘Jean’), these results are following the tartaric acid concentration that ranged from 2.49 to 7.70 g/L of tartaric acid. In another study with red grapes from the Douro and Dão regions, Portugal, it was found that red grapes from the Douro Region had a higher content of tartaric acid (average values 6.21 g/L) compared to red grapes from Dão (4.96 g/L) [41].

### 2.3. Dietary Fibers

There are several definitions of dietary fibers [46], according to the Institute of Medicine, [47], the term dietary fiber consists of non-digestible carbohydrates and lignin that are intrinsic and intact in plants. The term includes cellulose, hemicellulose, lignin, pectins, gums, mucilages, and a non-carbohydrate component [1]. Anita and Abraham [48] classified the dietary fiber into two categories: cellulose, hemicellulose, and lignin, which are water-insoluble/less fermented, being the pectins, gums, and mucilages water-soluble/well-fermented fibers.

The benefits of a high fiber diet have long been known [49], and we are always incited to eat fiber-rich foods [50]. Thus, in addition to the direct consumption of fiber-rich products and ingredients, the food industry has been searching for new sources of dietary fiber and developing new products with fiber supplementation [51]. Diets rich in fruits with high content of fibers have been related to the decreased incidence of several types of diseases [52]. Indeed, the modulation of function of the intestinal tract [50], lower risk of colorectal cancer [52], reduction in the total and LDL cholesterol [52] and cardiovascular disease, reduction onset risk or symptoms of metabolic syndrome and type 2 diabetes, are benefits of a fiber-rich diet. In addition, these types of diet are usually relatively low in calories compared to meals rich in other food types [53]. It is recommended that adults should ingest about 20 to 35 g of dietary fiber per day [54]. The fiber content of several red fruits is present in Table 4. Among red fruits, the cranberry presents a higher content in fiber followed by the raspberries and blackberries. Strawberries and cherries appear to be the least rich in fiber, with values between 1.3 and 2.2 mg/100 g FW. According to Akimov et al. [55], insoluble nutritional fiber is dominant in raspberry (68.6%). The necessary daily norm for the consumption of nutritional fiber is 20 g [54]. One hundred grams of raspberry fruit provides the human organism with 11.7% of such components.

### 2.4. Lipids and Fatty Acids

The increased consumption of red fruits is associated with their nutritional value, which offers many health benefits, particularly in the prevention of cardiovascular diseases and reduction in cancer risks. Red fruits, when consumed in the raw form, present reduced levels of fat, generally below 1% (Table 5). Pacifico et al. [59] studying two sweet cherry cultivars (‘Del Monte’ and ‘Della Recca’) found differences in their lipid composition. Similar findings were described by Kafkas et al. [60] in nine strawberry cultivars (‘Call-Giant4′, ‘Camarosa’, ‘Fern’, ‘Festival’, ‘Kabarla’, ‘Redlans Hope’, ‘Sweet Charlie’, ‘Whitney’, and ‘Gianna’) of Turkey. Kafkas et al. [61] in seven raspberry cultivars (‘Heritage’, ‘Canby’, ‘Willamette’, ‘Hollanda Boduru’, ‘Newburgh’, ‘Tulameen’, and ‘Meeker’) and Fadavi et al. [62] in 25 pomegranates varieties grown in Iran also found differences in the lipid percentage and fatty acid composition. According to Al Juhaimi et al. [63], the fatty composition of grapes is dependent on the local origin. The same conclusion was achieved by Melgarejo and Artés [64] when comparing the oil content and fatty acid composition of the oilseed of seven sweet Spanish varieties with some Oriental pomegranate varieties. Al-Maiman and Ahmad [65] also observed a slight modification in lipid and fatty acid content during different stages of pomegranate maturation (unripe fruits, half-ripe fruits, and full-ripe fruits). Wang and Wang [66] analyzing the effect of storage conditions on several cranberries’ varieties and concluded that storage temperature greatly affected their fatty acid profile. The types of product processed, as well as its residues, also affect the lipid content and the fatty acid composition. Recently, Zafra-Rojas et al. [67] found that blackberry residues comprised of peel, seeds, and pulp exhibited lower content of fatty acids when compared with the commercial product.

The lipid fraction of red fruits is rich in unsaturated fatty acids, particularly polyunsaturated fats (Table 6). The berry fruits (raspberry, strawberry, blackberry, and blueberry) are a remarkably good source of polyunsaturated fats (Table 6), with a significant amount of linoleic (C18:2, n-6), and linolenic (C18:3, n-3) fatty acids. Red fruits also contain considerable amounts of oleic acid (C18:1, n-9), palmitic acid (C16:0), α-linolenic acid (C18:3, n-3), stearic acid (C18:0), and myristic acid (C14:0). Contrary to all red fruits considered in this review, lipid fraction in pomegranate seeds consist mainly of punicic acid (C18:3, n-5), a conjugated isomer of α-linolenic acid [69].

Lipids present in fruits are crucial in the metabolism since they are included in cell membranes, increase resistance to viral infections and catarrhal diseases [78]. In red fruits, the low lipid content associated with low cholesterol levels makes these fruits highly appreciated by consumers. Nowadays, consumers have increasingly been more concerned with the nutritional and caloric value of the food they consume, looking for healthier, innovative, safe, and easy-to-use products. Therefore, the consumption of red fruits has increased, in part due to an array of health benefits. Additionally, they present some type of fatty acids with numerous positive effects on human health, as an anticancer and neuroprotective agent, is also associated with cardiovascular diseases protection [79]. Fatty acids in red fruits are mainly in the form of polyunsaturated acids, which are necessary to build cell membranes and the covering of nerves as well as for proper blood clotting, muscle movement, and protection against inflammation [80]. Polyunsaturated fatty acids are essential for normal body functions and are associated with significant beneficial cardiovascular effects [81].

According to the Food and Agriculture Organization/World Health Organization, about 2–4% of daily energy should come in the form of essential fatty acids with an additional 3% energy for pregnant or breastfeeding mothers [82]. The dominant fatty acids found in red fruits are omega-6 linoleic acid, omega-9 oleic acid, and omega-3 linolenic acid, which are linked to an array of health benefits. Omega-3 and omega-6 are crucial for the prevention and treatment of cardiovascular disease [81]. Their incorporation in a diet can result in a decrease in mortality from coronary artery and cardiovascular diseases [81]. Still linked to the reduction in cardiovascular disorders is omega-9, the most common monounsaturated fatty acid, which has also been linked to beneficial effects for diabetes [83].

### 2.5. Polyphenols

#### 2.5.1. Phenolic Acids and Flavonoids

**Figure 2:**
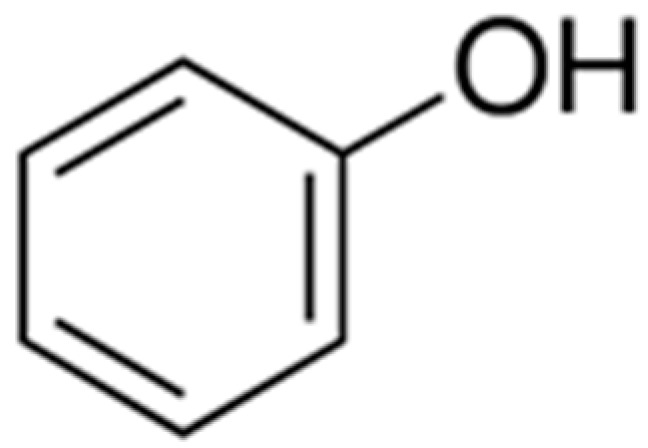
The basic carbon skeleton of phenolics.

Phenolic compounds are ubiquitous in plants, and as in other types of fruits they are present in high amounts in red fruits. They are one of the most studied secondary metabolites due to their bioactive functions such as anti-proliferative, anti-diabetic, anticancer, anti-microbial, anti-inflammatory, and antiviral, along with their high antioxidant capacity [84]. Phenolics are a group of hydroxylated molecules gathered in different types of structures with a common aromatic ring (Figure 2), and currently, about 8000 different structures of plant phenolics are known [85].

**Figure 3:**
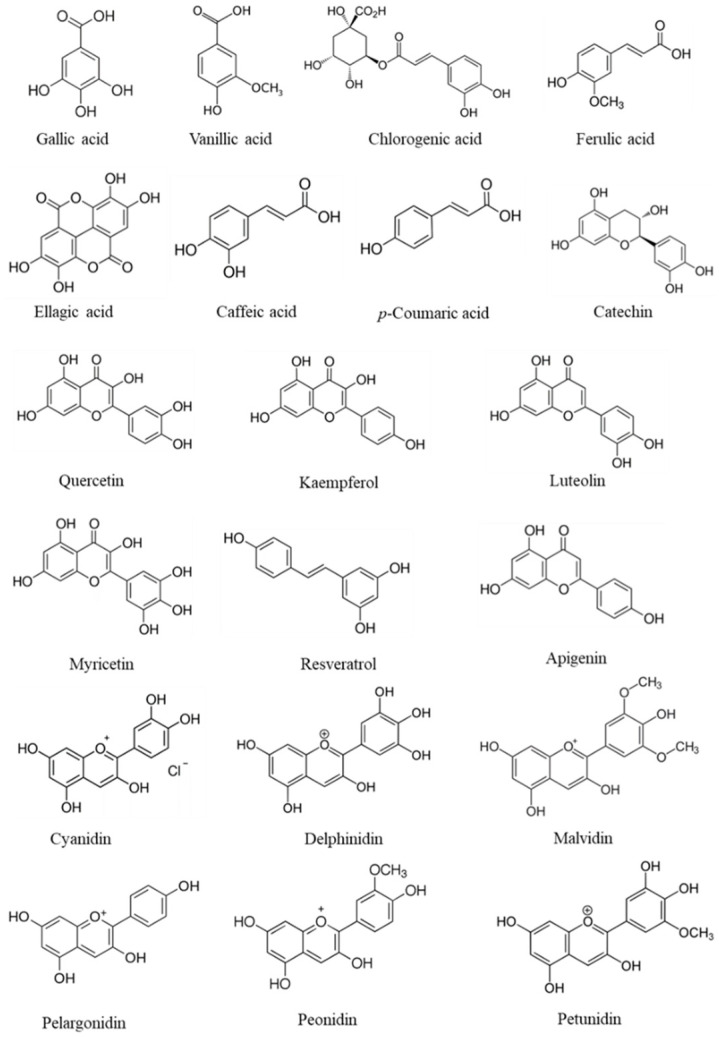
Chemical structures of phenolics that are commonly found in fruits.

There are many criteria to classify or distinguish phenolics, however, the most commonly used is the division of phenolics into flavonoids and non-flavonoids. According to Działo et al. [85], flavonoids are based on two aromatic rings connected by a bridge consisting of three carbons (C6-C3-C6), which are divided into six main sub-classes: flavonols, flavones, flavanones, flavan-3-ols, isoflavones, and anthocyanidins. In nature, flavonoids occur usually in association with sugar as glycosides. The second class of phenolics is the non-flavonoid molecules [85], which can be divided into 3 subgroups: phenolic acids, lignans (C6-C3)2, and stilbenes C6-C2-C6 [86]. Phenolic acids can be divided into two categories: hydroxybenzoic (C6-C1) acid derivatives and hydroxycinnamic (C6-C3) acid derivatives. The first group includes molecules such as hydroxybenzoic, gallic, vanillic, and ellagic acid, and in the second group *p*-coumaric, caffeic, ferulic, and chlorogenic acids are the most representative [87]. Flavonoids include catechin, quercetin, kaempferol, luteolin, and myricetin, as the most relevant compounds [85,87,88]. Anthocyanidins include cyanidin, delphinidin, malvidin, pelargonidin, peonidin, and petunidin as the most representative molecules [87,89]. Figure 3, illustrates the chemical structures of the most common phenolics found in fruits.

According to the literature, in red fruits diverse and high amounts of phenolics can be found; however, each type of fruit contains a typical phenolic profile. Table 7 summarizes the most common type of red fruits and the main type of phenolics often associated with them. Certain similarities in the phenolic profile can be observed within the same plant genera.

In general, the majority of phenolics present in red fruit belong to the main classes: phenolic acids and anthocyanins; nonetheless, it is possible to find other types even if they are at lower concentrations. Table 8 and Table 9, shows the average levels of different types of phenolic acids and flavonoids often found in red fruits.

Certain similarities in phenolic content can be observed within the same plant family and genus. For example, flavonols and hydroxycinnamic acids are typical of the Ericaceae family, genus *Vaccinium* (bilberry, blueberry, cranberry, and lingonberry) [90,92,94,95,96], while flavonols dominate in gooseberry, black and red currant (Grossulariaceae family, genus *Ribes*) [98,101]. Instead, ellagic acid is the main phenolic compound in fruits from the Rosaceae family, genus *Fragaria* and *Rubus* (strawberry and red raspberry) [93,101,102].

Despite the similarity between red fruits, each species has its typical profile. For example, Häkkinen et al. [111] reported that blueberry is rich in quercetin and caffeic acid, while bilberry and lingonberry have residual concentrations of quercetin. Hydroxycinnamates dominated in all cherry samples and represented 60–74% by weight of the phenols in the fresh and stored samples of the varieties ‘Saco’, ‘Summit’, and ‘Van’, and 45% by weight of the phenols in the cv. Burlat samples, which were richer in anthocyanins. The relative and total levels of hydroxycinnamates, anthocyanins, flavonols, and flavan-3-ols varied among sweet cherry cultivars and during storage. Moreover, cold storage induced decreased total phenol levels in the varieties ‘Summit’ and ‘Van´, however increased total phenol levels in the varieties ‘Burlat’ and ‘Saco’ [112,113].

#### 2.5.2. Anthocyanins

Anthocyanins are generally accepted as the largest and most important group of phenolics in red fruits. Anthocyanins are water-soluble compounds responsible for the blue, purple, red, or black color of many fruits, including red fruits. Until now, there are about 17 anthocyanidins properly identified in nature, however only six of them, cyanidin, delphinidin, petunidin, peonidin, pelargonidin, and malvidin, are found in most foods and plants [89]. When anthocyanidins are coupled with sugars, anthocyanins are formed [87]. Table 10 shows some average levels of the main anthocyanins most common in red fruits.

Cyanidin-3-*O*-rutinoside and cyanidin-3-*O*-xylosylrutinoside were the main anthocyanins found in black raspberries [117], cyanidin-3-*O*-rutinoside and cyanidin-3-*O*-glucoside were the main anthocyanins found in sweet cherry [112,118], and delphinidin-3-*O*-galactoside was the main anthocyanin in blueberries [119]. The application of gibberellic acid, abscisic acid, and glycine-betaine at pre-harvest increased anthocyanin content in cherries [120]. In red raspberry cyanidin-3-*O*-sophoroside and cyanidin-3-*O*-glucoside were the main phenolics found [16,110,121]. Guiné et al. [93] found that red raspberry was also rich in ferulic acid, vanillic acid, and delphinidin-3-*O*-glucoside, while gooseberry was rich in chlorogenic acid and cyanidin-3-*O*-glucoside. Strawberry was shown to be rich in pelargonidin and ellagic acid [110,122].

Grapes are the main dietary source of anthocyanins. They are considered to have diverse biological properties and therefore are regarded as secondary metabolites with potential nutritional value. Anthocyanins are mainly localized in berry skin. Grape anthocyanins are the 3-*O*-monoglucosides of delphinidin, cyanidin, petunidin, peonidin, and malvidin. Glucosylated derivatives of these anthocyanins, esterified at the C6 position of glucose with acetyl or coumaroyl groups have also been found, albeit at low concentrations. The monomeric anthocyanins in grape skin extracts were mainly malvidin (1.40–7.09 mg/g of skin), in particular, malvidin-3-glucoside (0.62–6.09 mg/g of skin) [40].

In cherry, extracts from stems of cv. Lapins and kernels of cv. Early Bigi presented high levels of total phenolics, flavonoids, and *ortho*-diphenols. In general, major phenolic compounds identified in stems and kernels were sakuranetin and catechin, respectively. Moreover, antioxidant activities showed a positive correlation with the increments in phenolic compounds [130].

During production, the correct combination of scion × rootstock can produce fruits with higher firmness, weight, sugars, vitamins, and phenolic compounds that boost the fruit’s antioxidant activity. Orchard management, such as applying drip irrigation and summer pruning, can also increase the total phenolic content, while application of growth regulators can result in improved storability, increased red coloring, increased fruit size, and reduced cracking. Salicylic acid, oxalic acid, acetylsalicylic acid, and methyl salicylate are promising growth regulators as they also increase total phenolics, anthocyanins, and induce the higher activity of antioxidant enzymes [131]. The application of biostimulants, such as glycine-betaine and *Ascophyllum nodosum* extracts increased polyphenols content and antioxidant capacity in cherries [132,133]. In grape, it was also observed that the application of chitosan on the whole vine before and after veraison led to the increased concentration of total phenolic compounds, anthocyanins, and tannins [134]. In this study, it was also observed that chitosan application not only induced the synthesis of phenolic compounds but also acted as a facilitator of phenolic transfer from leaves towards grape berries. Furthermore, the application of other foliar mitigation treatments (kaolin (5%) and potassium silicates (0.1 and 0.05%)) influenced the grape berry quality, namely the concentration of total anthocyanins and monomeric anthocyanins [135].

The tannin profiles of five grapes (*Vitis vinifera* L.) varieties were studied by Cosme et al. [136]. Depending on the grape variety, the polymeric fractions in skins represented 91–99% and the distribution of the mean degree of polymerization (mDP) of the skin proanthocyanidins ranged from 3.8 to 81.0.

According to data presented in Table 7, Table 8, Table 9 and Table 10, and many others reported in the literature, anthocyanins are the most important group of phenolics in red fruits, while flavonoids and phenolic acids are present in lower amounts. The most abundant anthocyanins are cyanidin and delphinidin, and in flavonoids are quercetin, kaempferol, and myricetin. Caffeic acid, *p*-coumaric acid, and ferulic acid are the most common phenolic acids found in red fruits.

Although it is common to find this profile in red fruits, the differences in their levels often found within the same genus and species are due to genotype, cultural practices, environmental conditions, fruit ripeness, and postharvest and storage conditions. Howard et al. [137] found that total phenolics, hydroxycinnamic acids, and anthocyanidins in blueberries can vary significantly between genotypes and between growing seasons, and from their point of view different genotypes should be always screened over multiple seasons to identify phenolic-rich germplasm. Similar results were achieved by Nour et al. [99] when they studied the variation in anthocyanins profile and content along with different genotypes of blackcurrant, and they report that genotype is one factor that significantly influences the content of phenolics. In addition, Vagiri et al. [98] found a significant variation in phenolics in blackcurrant due to genotypes, ontogenic stage, and location. In 2012, Aaby et al. [102] found a significant variation in phenolic profile and content in 27 cultivars of strawberries during ripening, which is a crucial stage of fruit development and has a crucial role in phenolic accumulation. Zhang et al. [138] studying the effect of maturation in the accumulation of anthocyanins in strawberry (*Fragaria* × *ananassa* Duch.), found that the major groups of compounds such as anthocyanins, amino acids, and sugars alter during growth and maturation. The same authors concluded that each stage of strawberry development has its unique metabolic profile, with the most drastic changes occurring at the transition toward the red-ripened stage. Beekwilder et al. [139] reported an increment in the level of anthocyanins during raspberry fruit ripening, while the levels of ellagitannins and proanthocyanidins decreased in the same period, probably due to differences in enzymatic activity of glycosyltransferases, which interfere in biosynthetic pathways of anthocyanins. Atkinson et al. [140] also found that cultural practices could interfere significantly with the content of phenolics. They found that using mulches in strawberry cultivation can increase the concentration of ellagic acid and ascorbic acid due to significant increments in light reflection. Vyas et al. [141] showed that environmental conditions had significant effects on the concentration of anthocyanins and proanthocyanidins in lingonberry, and the levels of these two types of antioxidant compounds were positively correlated with latitude, altitude, reduced temperature, and increased precipitation of the collection sites.

Another important set of factors that affects the content and availability of phenolics in red fruits are the post-harvest factors, which include the storage conditions and processing. Kozos et al. [142] found that storing blueberries in a controlled atmosphere for six weeks decreases the loss of anthocyanins compared to a normal atmosphere. The same authors found that to preserve the high quality of blueberries, the fruits must be cooled quickly after harvest and stored in a cold room with a controlled atmosphere. Also, Srivastava et al. [143] observed retention higher than 40% in ellagic acid and quercetin, two important antioxidant phenolics, when blueberries were stored at 1 °C for 35 days. Mullen et al. [144] observed an increment in ellagitannins of red raspberries when they were stored at 4 °C for 3 days. In addition, Ayala-Zavala [145] found that temperatures of 0 °C retained higher contents of antioxidant compounds in strawberries for longer periods than those stored at 5 °C or 10 °C.

The loss of phenolic content in fruits are expected when they are converted into juices or jams, due to the complexity of processing steps involved. In general, when fruits are converted into juices, a frozen or refrigeration step is followed by blanching, milling, depectinization, pressing, pasteurization, and if necessary clarification and concentration. All these steps can play a major role in phenolic degradation. For example, anthocyanin retention in cranberry juice is generally lower than 50% due to losses during various stages of processing [146]. Buchert et al. [147], comparing the effects of the usage of different enzyme preparations on anthocyanin composition of bilberry and blackcurrant juices during the juice preparation, observed that the enzyme activities of β-galactosidase, α-arabinosidase, and β-glucosidase varied significantly, and the presence of β-galactosidase resulted in complete loss of delphinidin, cyanidin, petunidin and malvidin galactosides in the juice. They also observed that the pressing step resulted in marked losses of anthocyanins due to physical removal of the anthocyanin-rich skins as well as the anthocyanins binding to cell wall polysaccharides. Furthermore, another study reported that frozen highbush blueberries (*Vaccinium corymbosum*) [148] showed a pronounced deterioration of phenolic compounds when frozen blueberries were processed into juice and concentrate. Nonetheless, Lee et al. [149] observed an increment of malvidin-glycosides and total anthocyanins increased by 51% and 60%, respectively, in pasteurized juices and concentrates compared to the fresh fruit, probably caused by the step of concentration. However, they noted that delphinidin-glycosides with pasteurization decreased by 26%. Pasteurization was found to exert a significantly destructive effect on the anthocyanin content of strawberry juice in 60–70% [150] due to the high temperatures used in this processing step. In jams, another way to process fruits the tendencies are similar to those observed in juices. Kim and Padilla-Zakour [151], studying the processing effect on phenolics and antioxidant capacity in anthocyanin-rich cherry, plum, and raspberry, found that the processing steps and heating during jam making decreased the contents of total phenolics by 48% for cherry, 57% for plum and 36% for raspberry. The content of total anthocyanins decreased 86% in cherry, 84% in plum, and 45% in raspberry [151]. Jam making generally involves fruit tissues disruption followed by heating under a high acidic environment to avoid any bacterial contaminations, resulting in considerable losses of anthocyanins [152]. However, some phenolics such as ellagic acid can increase between 4.6 fold in black raspberry [152] and 1.5 to 2.5 fold during the processing of raspberry [153] and strawberry [154] jams. This increase may be correlated to an easier extractability of ellagic acid in this heat and high acid environment [151].

Despite these variations, numerous studies demonstrated that various phytochemical constituents of red fruits exhibit a wide range of biological effects [84,155], including antioxidant activities, which will be addressed in point three of this work.

### 2.6. Aroma and Flavor Compounds

Due to the increased interest in the health benefits of small berry fruits, research in berries’ flavor quality has also increased. Volatiles found in small berries are diverse and give unique flavors to different fruits. Fruit flavor also depends upon taste (sweetness and sourness, and low or no astringency) and aroma (concentration of volatile organic compounds). Nevertheless, while the environment may alter the flavor quality of small fruits, genetic factors seem to determine the flavor profile quantitatively and qualitatively [156].

Strawberries (*Fragaria* × *ananassa*) are among the most appreciated fruits worldwide. The modern garden-strawberries varieties present largeness, beautiful red color, and extended shelf life. However, the sensory quality is often censured by consumers, as they seem deficient in flavor and fragrance [157].

Numerous volatiles, of several chemical natures, have been identified in ripe strawberries including alcohols, aldehydes, esters, furanones, ketones, and terpenes [158]. Wild strawberries, opposite to garden varieties, present intense flavor, and fragrance [159]. These varieties provide an appreciated source of volatile compounds for breeding new commercial strawberries with improved aroma [160]. One example is *Fragaria moschata* or musk strawberries, which are recognized for their well-known aroma.

At present, few musk strawberries survive in farm plantings. One example is the Italian clone ‘Profumata di Tortona’, considered one of the most fragrant strawberries [161]. The berries of this variety are characterized by an intense red color, a pale flesh, a delightful sour sweet taste, and astringent flavor, presenting aromas of caramel, mango, and tropical-fruits scent [158,162].

Ulrich and Olbricht [163,164] identified significant differences in the presence of individual esters, ketones, and terpenes between *Fragaria* × *vesca* samples and *Fragaria* × *ananassa* cultivars. Mainly in esters (ethyl hexanoate, methyl butanoate, and methyl hexanoate) that were found in higher concentrations in *Fragaria* × *ananassa* compared with wild samples. Contrariwise, the ester methyl anthranilate (2-aminobenzoic acid methyl ester), the aroma compound associated with strawberry aroma/flavor [165], presents the characteristic fragrance of wild strawberries with a fruity, concord grape, musty with a floral powdery nuance, was more abundant in *Fragaria* × *vesca*. Likewise, ketones (2-pentanone, 2-heptanone, and 2-nonanone) and terpenes (myrtenal, myrtenil acetate, α-terpineol) are present in higher levels in *F. vesca*, excepting the monoterpene linalool, more abundant in cultivated strawberries.

**Figure 4:**
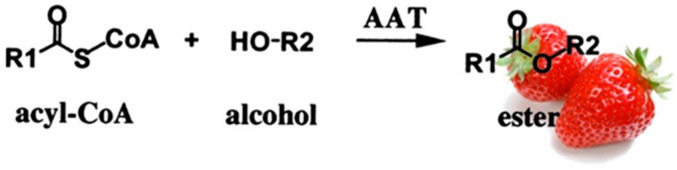
Schematic representation of the esterification reaction catalyzed by the alcohol acyltransferase (ATT).

As it was mentioned before, esters are important volatile compounds in fruit flavor, and in strawberry, several esters have been identified [166]. The reaction of transacylation from acyl-CoA into alcohol is called esterification (Figure 4). The enzyme that catalyses the reaction is alcohol acyltransferase (AAT). Ueda et al. [167] concluded that, in strawberry, the alcohol moieties of the esters produced revealed the alcohols mainly synthesized in the fruit, and the acid moieties reflected the acyl-CoA specificity of the AAT enzyme. The strawberry AAT enzyme had a high activity with hexanol and with acetyl- or butyl-CoAs [168,169].

Recently, Negri et al. [159] identified 131 volatile compounds in ripe berries of ‘Profumata di Tortona’ and *F. vesca* cv. ‘Regina delle Valli’, a number exceeding the aroma compounds usually found in commercial strawberries. Moreover, 80 volatile compounds have been already identified by Schwieterman et al. [170] in 35 strawberry-garden varieties. In total, and according to Ulrich et al. [171], 979 volatile compounds were identified, 590 of which were found since 1997.

**Figure 5:**
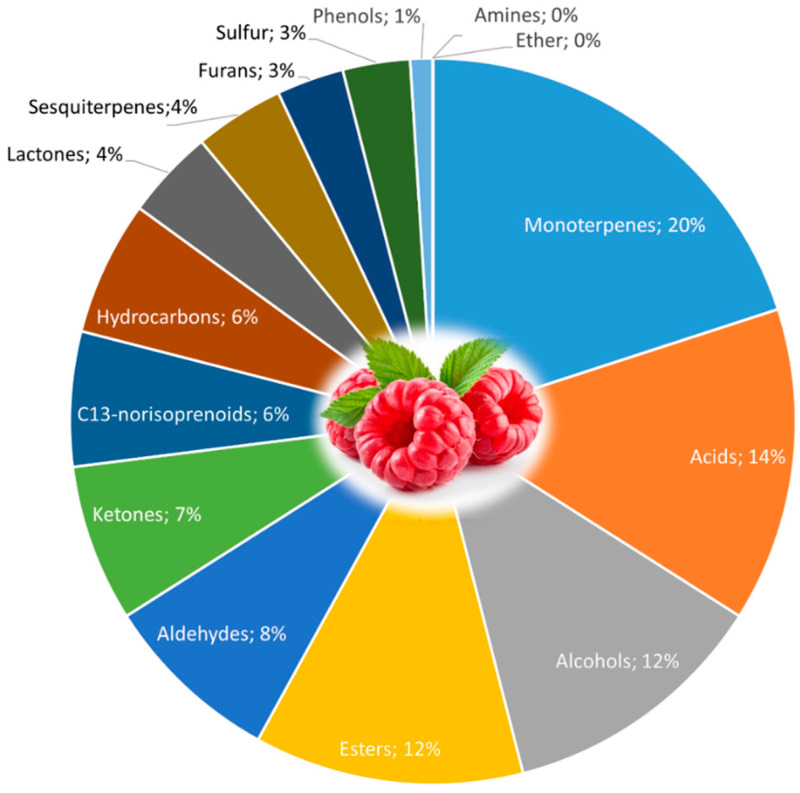
Volatile compounds in raspberry fruit (*Rubus idaeus* L.) and their chemical class. Adapted from [172].

Raspberry volatiles are important for the perception of their sensory quality and mold resistance [172], Figure 5. As to other fruits, volatile compounds are influenced by numerous factors including ripeness, climate, soil, cultivar variation, among others [173].

The volatile compounds in raspberry are mostly free forms of different metabolites, most of them present in the form of glycoside bound to sugars, able to release free volatile compounds by enzymatic or chemical cleavage, the process that can occur during plant maturation, industrial treatments, and fruits processing [174].

Honkanen et al., in 1980 [175], identified a total of 75 volatile components in raspberry oil in the pressed juice. More than 40 were not reported previously. These comprised 2,5-dimethyl-4-methoxy-3(2H) furanone, 5-methyl-4-hydroxy-3(2H) furanone, 2,5-dimethyl-4-hydroxy-3(2H) furanone, terpenes, and a few esters including ethyl 5-hydroxyoctanoate and ethyl 5-hydroxydecanoate, that had not been identified in natural products [175].

The aroma of blackberry fruits affects the quality of either fresh or processed fruits. The volatile compounds responsible for the fruit flavor are biosynthesized through metabolic pathways during ripening, harvest, post-harvest, and storage periods. According to Georgilopoulos and Gallois [176], it seems that the aroma of blackberry juice is mainly due to the presence of furfural, 3-methyl-butanal, 3-methyl-1-butanol, phenylacetaldehyde, and *trans*-furan linalool oxide. However, Du and Qian [156] state that the most potentially important odor-active compounds in ‘Black Diamond’, a blackberry variety, are ethyl hexanoate, ethyl butanoate, 1-octen-3-one, 2-heptanol, *cis*-3-hexenol, linalool, nonanal, *trans*-2-hexenol, methional, ethyl 3-hydroxyhexanoate, α-ionone, β-ionone, furaneol, and 5-isoprenyl-2-dimethyl-divinyltetrahydrofuran.

Blueberry aroma depends on the interaction of dozens of volatile compounds [177]. Most research has been conducted, in recent years, with the aim of understanding the full complexity of blueberry flavor. For instance, Beaulieu et al., in 2014 [178], when studying five Louisiana-grown RAB cultivars (‘Brightwell’, ‘Climax’, ‘Premier’, ‘Powder Blue’, and ‘Tifblue’) found compounds such as linalool, methyl 3-methylbutanoate, 1,8-cineole, (*E*)-2-hexanal, (*Z*)-3-hexenal, (*Z*)-3-hexenyl acetate, limonene, hexyl acetate, hexanal, and α-terpineol. More recently, Farneti et al. [179] studied the intricacy of blueberry aroma by untargeted volatile organic compounds analysis, performed by solid-phase microextraction- gas chromatography-mass spectrometry (SPME-GC-MS) and proton transfer reaction—time of flight—mass spectrometry (PTR-ToF-MS). They found that the compounds usually considered responsible for blueberry aroma are synthesized by the fruit in the ripe stage. They are monoterpenes (such as linalool), (*Z*)-2-hexen-1-ol, and hexanal. Several compounds can be detected in fruits at the pink stage of ripening like the (*E*)-2-hexenal.

Hirvi et al., in 1981 [180], found a huge number of volatile compounds in cranberry samples. A total of 70 components were identified in cranberries from two continents, Europe and America. The aroma of cranberries is characterized by the presence of several aromatic metabolites together with α-terpineol (terpinolene, *p*-mentha-1,8-diene, thujene, *p*-mentha-1,8-diol, carvenone, 1-phenylethanol, 3-phenylpropan-1-ol, *trans*-cinnamyl alcohol, 2-(4-hydroxyphenyl)-ethanol, 2-(4-methoxyphenyl) ethanol, salicylaldehyde, 4-methoxybenzaldehyde, 3-phenylpropanal, *trans*-cinnamaldehyde, vanillin, 4-hydroxyacetophenone, 4-methoxyacetophenone, 4-(4-hydroxphenyl) butan-2-one, 4-(4-hydroxy-3-methoxyphenyl) butan-2-one, methyl cinnamate, 2-phenylethyl formate, and some γ-and α-lactones. Nevertheless, not all these compounds are aromatic in the sense that they can be perceived by the human nose.

When considering the potential contribution of a volatile compound to the aroma of fruit, the determination of the odor-activity value (OAV) and odor detection threshold is of extreme importance. An OAV is used to estimate odor potency in terms of the ratio of the concentration of a volatile compound to its odor detection threshold. In 2016, Zhu et al. [181] identified several compounds and also studied their OAVs. They found that the hexanal, pentanal, (*E*)-2-heptenal, (*E*)-2-hexenal, (*E*)-2-octenal, (*E*)-2-nonenal, ethyl 2-methylbutyrate, β-ionone, 2-methylbutyric acid, and octanal contributed greatly to the cranberry aroma. So, we can say that the list proposed by Zhu et al. [181], presents the true form of cranberry aroma/flavor.

## 3. Red Fruits: Consumers’ Perception of Health Benefits

The consumer’s quality perception can be influenced by the fruit’s intrinsic attributes, but also by extrinsic indicators provided by the seller of the fruit; like brands, product origin, or quality labels [182]. According to Grunert [183], consumers are interested in the benefits of fruit consumption and how it can help them attain their values. This means that consumers, besides judging the fruit quality, also ponder if the specific fruit properties are desirable and likely to fulfill their preferences and needs by linking the fruit characteristics to self-relevant consequences and personal values [184]. Recent studies have mentioned pleasure-seeking as the primary motivation for fruit consumption [185]. People seem to enjoy looking at, handling and selecting the fruits they want to eat. Freshness, firmness, size, color, and flavor are important attributes for fruit selection, and visually attractive fruits are likely to act as a promoter of purchase and consumption intentions [186]. Although overall fruit quality is the key factor behind purchase decisions [187], the price cannot be discarded as part of the decision process by consumers [188,189]. Availability, packaging, convenience, and brand are other search determinants often cited in the literature to affect consumer food choices [190,191,192,193]. Furthermore, consumers still see a link between healthier food and less-than-optimal sensory attributes [185,194], which can lead to a lower intake of healthier foods, although some consumer segments characterized by older people and women are willing to trade taste for health benefits [195].

Plant science research has been primarily focused on the increase in production, with health benefits being a minor concern. However, consumers’ awareness of the health effect of fruit intake against chronic diseases makes this a major topic for discussion and research [196]. The food industry is currently adapting its market trends to accommodate sustainability values, especially those related to health benefits, as they are increasingly searched by the consumers [197], based, for fruits, on the phytonutrients present in these foods [196].

The current use of phytonutrients by food producers and the knowledge of their effect on the prevention of chronic disease points out the need for a careful look at crop production strategies (fertilization, season, soil fertility, and irrigation) affecting the quantitative and qualitative profiles of these compounds, but also to post-harvest techniques (processing or packaging) that can modify phytonutrients [196]. There is mounting evidence of the potential health benefits of a fruit-rich diet. The ingestion of phytochemicals from fruits and their positive influence on several diseases (cancer, heart disease, stroke, hypertension, birth defects, cataracts, diabetes, diverticulosis, and obesity) were established [197,198,199]. There are many phytochemicals present in fruits that can be responsible for their health-promoting activities. Vitamins, carotenoids, phenolic acids, trihydroxystilbenes, or flavonoids, are often associated with the prevention of certain cancers and cardiovascular diseases, but also phytoestrogens, organosulfur compounds, fiber, or isothiocyanates (reviewed by [200,201]). The majority of small fruits and grapes have a high antioxidant activity, and present in their composition several compounds of interest [202,203].

In cherry, phenolic extracts inhibited low-density lipoprotein oxidation in vitro in a dose-dependent manner. Extracts of freshly harvested cherries exhibited significantly higher antioxidant activities than extracts of stored samples at cold temperature. The cv. Summit samples had the highest antioxidant activity. Differences in the antioxidant effects of the cherry samples were positively correlated with their levels of *p*-coumaroylquinic acid (*p* < 0.1) but negatively correlated with their cyanidin-3-*O*-rutinoside levels (*p* < 0.05) [113].

**Figure 6:**
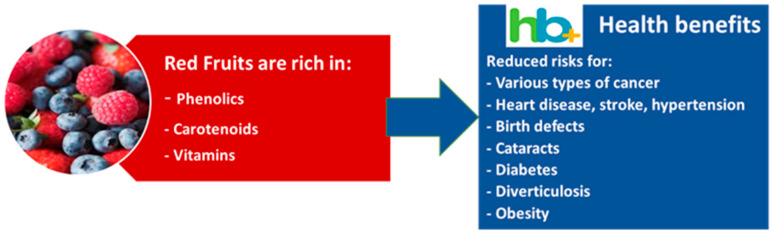
Health effects of red fruits.

There are still many studies to be conducted for the advancement of functional foods into the diet routines of consumers. Although there has been a strong emphasis on showing how fruits can prevent diseases and increase health, rather than just providing nutrients, further research has to focus on how to effectively inform consumers of nutrition and health properties. For instance, although it appears that 87% of Americans believe that certain foods have health benefits, with fruits recognized as the top food category with health benefits [204], other authors point out the lack of awareness of consumers to such facts [205]. An increase in consumers’ knowledge of the health benefits of fruit consumption will, most likely, increase the intake of fruits, allowing their long-term marketplace success [206], with clear benefits, not only for the consumer’s health but also for producers and sellers. Some works point out that an increase in the public awareness of the link between diet and health can be responsible for higher per capita consumption of fruits [201] (see Figure 6).

In the past few years, many studies have shown that phenolics and other molecules present in red fruits have different bioactive properties, including antioxidant activity. The antioxidant activity of red fruits and their bioactive molecules, including phenolics have been assessed using in vitro assays, measuring its ability to reduce and trap free radicals and reactive oxidant species (ROS) produced from a wide range of physiological processes. Different methods such as oxygen radical absorbance capacity (ORAC), 2,2′-azinobis-(3-ethylbenzothiazoline-6-sulfonate (ABTS), 2,2-diphenyl-1-picrylhydrazyl (DPPH) or ferric reducing antioxidant power assay (FRAP) [155,207], among others, have been used to assess this capacity. The results from many authors have shown that red fruits have high levels of antioxidant activity due to their high content of phenolics and vitamin C, among other compounds. Some examples of the antioxidant capacity of red fruits are shown in Table 11.

These and other results from antioxidant activity assays performed by many authors have shown that red fruits can serve as a good natural source of bioactive and antioxidant molecules in the human diet, due to the high contents of anthocyanins, other flavonoids, and phenolic acids. For example, Tulipani et al. [217] found that eating strawberries (500 g every day) for 16 consecutive days, increased the plasma total antioxidant capacity (TAC) and the antihaemolytic defenses of human erythrocytes due to its high content of anthocyanins and vitamin C. Vasileiou et al. [218], reported that regular consumption of cranberries could prevent bacterial adherence to uroepithelial cells, which reduces the development of urinary tract infections, which could be related to its richness of phenolics, mainly anthocyanins. Riso et al. [219] found that the consumption of blueberries-based drinks with high content of anthocyanins for six weeks, reduced significantly the levels of oxidized DNA bases and increased the resistance to oxidatively induced DNA damage. In a study with 12 colored berries including black currants, blueberries, and red and black raspberries [220], it was found a strong correlation between the phenolic composition and the high levels of antioxidant activity. The same authors have concluded that phenolic components may be used directly to evaluate the potential health benefits of berries and other types of fruits.

Also, several diseases are related to a diet low in certain minerals. Low levels of potassium intake are reflected in higher blood pressure [221], higher risk of developing kidney stones since the acid-base metabolism is positively affected by potassium [222]. The heartbeat and muscle contraction are also affected by the level of potassium in the body [223]. Calcium is important for bones and tooth formation and decreases the risk of osteoporosis [224]. The magnesium levels in the body affect protein synthesis, muscle contraction, body temperature regulation, and the activation of moreover 100 enzymes [6]. Skeletal mineralization and some cellular functions such as phospholipid synthesis and intracellular regulatory, glycolysis, gluconeogenesis, DNA and RNA synthesis, cellular protein phosphorylation, depend on the presence of the inorganic phosphate [28]. On the other hand, the biosynthesis of amino acids is related to the presence of nitrogen. Even so, this mineral is also an essential component of nucleic acids, cofactors, and other metabolites [6].

Indeed, food choice is one of the most frequent human decisions and is determined by a complex set of factors and interrelated determinants [225]. Although several models attempting to explain that process have been proposed, one of the most accepted is the proposed Total Food Quality Model [226]. This model can be divided into three parameters: ‘search’, ‘experience’ and ‘credence’ attributes. The first two (search attributes, like appearance or price, and experience characteristics, like flavor or taste) are those more easily observed by consumers and can be straightforwardly experienced by them. For credence properties, like health and nutritional benefits, the consumer cannot validate those claims [227]. This must be where most effort should be employed; the increase in the consumers’ awareness of the health benefits of the intake of fruits, always considering the latest scientific progress and keeping in mind that these credence properties are a matter of credibility and trust that cannot be broken.

Hence, all encouraging actions that increase the intake in those fruits, and, therefore, of health-promoting compounds, are of great importance. This is even more important in our current society, where the fast food supply is large, widely available, and more easily responds to the fast-paced life of consumers, with nutritionally poor foods taking the place of a healthier diet.

## 4. Final Remarks

Fruits, especially small fruits, are rich in bioactive compounds such as vitamins and phenolic compounds, with well-demonstrated potential health benefits. However, we are at the beginning of acquiring knowledge to understand the variation in these compounds throughout the fruit development and the postharvest period. How climate change and innovative crop production technology affect plant performance, yield, and fruit quality is at the moment largely unknown. Further research should be aimed to develop species-specific strategies that improve both fruit quality and nutritional properties, without significantly affecting the yield. New fruit species and cultivars with improved traits are needed to produce fruits with excellent quality, and a high consumer appeal, that is well adapted to the different growing regions and future climate change.

## Figures and Tables

**Table 1:** The composition in Vitamin C (ascorbic acid) and other vitamins of some red fruits (units at the Table footnote).

Red Fruits							
	Sweet Cherry	Cranberry	Blackberry	Blueberry	Raspberry	Strawberry	References
	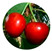	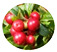	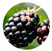	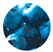	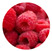	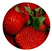	
Vitamin C (Ascorbic acid)	62.4 ^a^	10 ^b^	34–52 ^a^	10–100 ^a^	5–92.2 ^a^	5–90 ^a^	[2,19,23,24,25,26,27]
Vitamin B6 (Pyridoxine)	790 ^c^	606 ^c^		1999 ^c^		1744 ^c^	[28]
Vitamin B2 (Riboflavin)	247 ^c^	69 ^c^		216 ^c^		93 ^c^	[28]

Units: ^a^ mg/100 g Fresh weight (FW); ^b^ mg/100 g Dry weight (DW); ^c^ μg/g.

**Table 2:** Mineral content (mg/100 g FW) of some red fruits.

	Sweet Cherry	Blackberry	Blueberry	Raspberry	Strawberry	References
Phosphorus (P)	12.2	7–29	8.6	5.7	6.6	[2,30,31]
Potassium (K)	90.9	77–349	70.1	71.8	51.2	[2,30,31]
Calcium (Ca)	-	6–29	-	1.14	2.20	[2,30]
Magnesium (Mg)	12.2	6–44.8	4.9	15.9	8.78	[2,30,31]
Zinc (Zn)	0.69	0.07–0.44	0.13	0.37	0.13	[2,30,31]
Iron (Fe)	1.16	0.28–1.28	1.24	1.06	1.0	[2,30,31]

**Table 3:** The average content of main sugars and organic acids in different types of red fruits.

Red Fruits	Main Sugars	Main Organic Acids	References
Berries	Fructose (18.0–57.2 g/L) Glucose (22.2–50.0 g/L) Sucrose (0.2–5.1 g/L)	Citric acid (2.9–16.2 g/L) Malic acid (3.3–24.7 g/L)	[33]
Raspberry (*Rubus idaeus*)	Fructose 35–45% of total sugars Glucose 30–35% of total sugars Sucrose 30–35% of total sugars		[42]
Strawberry (*Fragaria* × *ananassa*)	Frutose (1.07–3.079 g/100 g) Glucose (2.236–4.802 g/100 g) Sucrose (0.352–7.571 g/100 g)	Citric acid (643.32 mg/100 mL) Malic acid (203.98 mg/100 mL)	[43,44]
Blueberry (*Vaccinium corymbosum*)	Fructose (70.40–304.52 mg/g DW) Glucose (13.86–57.36 mg/g DW) Sucrose (0.56–7.90 mg/g DW)	Citric acid (13.34–75.11 mg/g DW) Quinic acid (2.86–11.56 mg/g DW) Malic acid (1.02–7.21 mg/g DW)	[45]
Sweet cherry (*Prunus avium*)		Malic acid Oxalic acid Shikimic acid	[37]

**Table 4:** Dietary fiber of some red fruits.

Red Fruits							
	Sweet Cherry	Cranberry	Blackberry	Blueberry	Raspberry	Strawberry	References
Dietary fiber (mg/100 g FW)	2.1	35.7 ^c^	4.5–5.3	1.9–2.4	5.8–6.5	1.3–2.2	[2,30,31]
**Estimated Fiber Components ^a^**							
Serving Size ^a^	1 cup(138 g)		1 cup(144 g)	1 cup(148 g)	1 cup(123 g)	1 cup(152 g)	
Total (100 g)	2.2		5.3	2.8	6.5	2.0	[56,57,58]
Insoluble (100 g)	1.6		4.7	2.4	5.3	1.5	
Soluble (100 g)	0.6		0.6	0.3	1.2	0.5	
Pectin ^b^ (100 g)	0.7		1.4	0.8	1.6	0.7	

^a^ Based on commonly consumed fruit servings [57]. ^b^ Fruit pectin = mean 35% (range 20–40%) of total fiber [58].

**Table 5:** Total fat and fatty acids (g/100 g FW) composition of different red fruits in the raw form. Adapted from [68].

Red Fruits	Total Fat	Fatty Acids		
		Saturated	Monounsaturated	Polyunsaturated
Raspberry (*Rubus idaeus*)	0.65	0.019	0.064	0.375
Sweet cherry (*Prunus avium*)	0.20	0.038	0.049	0.052
Strawberry (*Fragaria* *×* *ananassa*)	0.30	0.015	0.043	0.155
Grapefruit, pink and red (*Citrus × paradisi*)	0.14	0.021	0.020	0.036
Cranberry (*Vaccinium oxycoccos*)	0.13	0.008	0.018	0.055
Pomegranate (*Punica granatum)*	1.17	0.120	0.093	0.079
Blackberry (*Rubus fruticosus*)	0.49	0.014	0.047	0.280
Blueberry (*Vaccinium corymbosum*)	0.33	0.028	0.047	0.146

**Table 6:** Predominant fatty acids in the seed oil of several red fruits.

Red Fruits	Main Fatty Acids	References
Sour cherry (*Prunus cerasus*)	Linoleic acid, oleic acid, palmitic acid, α-linolenic acid and stearic acid	[70]
Sweet cherry (*Prunus avium*)	Linoleic acid, oleic acid, palmitic acid, α-linolenic acid, and myristic acid	[71]
Strawberry (*Fragaria* × *ananassa*)	Linoleic acid, linolenic acid, oleic acid, palmitic acid and stearic acid	[61,72]
Grapefruit (*Citrus × paradisi*)	Linoleic acid, oleic acid, palmitic acid, stearic acid, and linolenic acid	[73]
Cranberry (*Vaccinium oxycoccos*)	Linoleic acid, oleic acid, linolenic acid, palmitic acid, and stearic acid	[74]
Pomegranate (*Punica granatum*)	Punicic acid, linoleic acid, oleic acid and palmitic acid	[75]
Raspberry (*Rubus idaeus*)	Linoleic acid, linolenic acid, oleic acid, palmitic acid and stearic acid	[72,76]
Blackberry (*Rubus fruticosus*)	Linoleic acid, α-linolenic acid, oleic acid, palmitic acid, and stearic acid	[77]
Blueberry (*Vaccinium corymbosum*)	Linoleic acid, α-linolenic acid, oleic acid, palmitic acid, and stearic acid	[77]

**Table 7:** Some examples of main polyphenols are found in different types of red fruits.

Red Fruits	Polyphenols	References
Bilberry (*Vaccinium mytillus*)	Delphinidin, cyanidin, petunidin, peonidin, malvidin, gallic acid, protocatechuic acid, 4-hydroxybenzoic acid, vanillic acid, caffeic acid, syringic acid, *p*-coumaric acid, ferulic acid, sinapic acid	[90,91]
Blueberry (*Vaccinium corymbosum*)	Cyanidin 3-*O*-glucoside, cyanidin 3-*O*-glucuronide, cyanidin 3-*O*-arabinoside, malvidin 3-acetylglucoside, malvidin 3-*O*-glucoside, peonidin-3-*O*-glucoside, petunidin 3-acetylglucoside, petunidin-3-O-glucoside, quercetin-3-*O*-rutinoside, quercetin-3-*O*-galactoside, quercetin-3-*O*-glucoside, kaempferol-3-*O*-glucoside, quercetin-3-*O*-diglycoside, quercetin 3-*O*-arabinoside, *trans*-5-caffeoylquinic acid, 3,5-dicaffeoylquinic acid, 4,5-dicaffeoylquinic acid	[92,93]
Cranberry (*Vaccinium oxycoccos*)	Cyanidin-3-*O*-galactoside, cyanidin-3-*O*-glucoside, cyanidin-3-*O*-arabinoside, peonidin-3-*O*-galactoside, peonidin-3-*O*-arabinoside, gallic acid, catechin, epicatechin, procyanidin A2 and *p*-coumaric acid, rutin, benzoic acid, caffeic acid	[94,95]
Lingonberry (*Vaccinium vitis-idaea*)	Cyanidin-3-*O*-galactoside, cyanidin-3-*O*-glucoside, cyanidin-3-*O*-arabinoside, proanthocyanidin A, proanthocyanidin B, ferulic acid, quercetin-3-*O*-glactoside, quercetin-3-*O*-glucoside, quercetin-3-*O*-arabinoside, quercetin-3-*O*-rhamnoside, kaempferol-pentoside, kaempferol-rhamnoside	[96]
Gooseberry (*Ribes uva-crispa*)	Cyanidin3-*O*-glucoside, petunidin-3-*O*-glucoside, pelargonidin chloride, caffeic acid, epigallocatechin gallate, p-coumaric acid, rutin, kaempferol, resveratrol	[93,97]
Black currant (*Ribes nigrum*)	Delphinidin-3-*O*-glucoside, cyanidin-3-*O*-glucoside, cyanidin-3-*O*-rutinoside, petunidin-3-*O*-rutinoside, pelargonidin-3-*O*-rutinoside, peonidin-3-*O*-rutinoside, epigallocatechin, catechin, epicatechin, neochlorogenic acid, chlorogenic acid, myricetin-malonylglucoside; quercetin-3-*O*-galactoside, quercetin-3-*O*-glucoside, quercetin-3-*O*-rutinoside, quercetin-3-6-malonylglucoside, kaempferol-3-*O*-glucoside, isorhamnetin-3-*O*-glucoside, kaempferol-malonylglucoside	[98,99,100]
Red currant (*Ribes pallidum*)	Cyanidin-3-*O*-glucoside, cyanidin-3-*O*-sophoroside, cyanidin-3-*O*-rutinoside, cyanidin-3-*O*-xylosylrutinoside, gallic acid, catechin, syringic acid, cinnamic acid, chlorogenic acid, ferulic acid	[100,101]
Red raspberry (*Rubus idaeus*)	Cyanidin-3-*O*-glucoside, delphinidin-3-*O*-glucoside, petunidin-3-*O*-glucoside, gallic acid, syringic acid, ferulic acid, quercetin	[93,101]
Strawberry (*Fragaria* × *ananassa*)	Cyanidin-3-*O*-glucoside, cyanidin-3-*O*-glucoside, cyanidin-3-*O*-malonylglucoside, pelargonidin-3-*O*-glucoisde, pelargonidin-3-*O*-rutinoside, pelargonidin-3-*O*-acetylglucoside, procyaniin dímers and pentamers, gallic acid, catechin, epicatechin, ferulic acid, *p*-coumaric acid, cinnamic acid, ellagic acid, quercetin-3-malonylglucoside, kaempferol-3-*O*-glucuronide, kaempferol-3-*O*-malonylglucoside	[102]
Crowberry (*Empetrum nigrum)*	Cyanidin-3-galactoside, chlorogenic acid, protocatechuic acid, batatasin-II, epicatechin, quercetin, kaempferol	[103]

**Table 8:** Example of phenolic acid contents found in the most common red fruits.

Red Fruits	Phenolic Acids (mg/kg FW)						References
	*p*-Coumaric	Caffeic	Chlorogenic	Ferulic	Gallic	Ellagic	
Bilberry (*Vaccinium mytillus*)	2.0–3.0	1.0–5.0	210.0–297.0	2.0–8.0	52.0–85.0	8.0–14.0	[104]
Blueberry (*Vaccinium corymbosum*)	n.d.-55.29	2.0–27.35	n.d.-700.0	9.60–22.0	n.d.-18.0	n.d.-1.0	[104,105]
Cranberry (*Vaccinium oxycoccos*)	n.d.	20.7–25.3	n.d.	60.5	n.d.	n.d.	[106]
Lingonberry (*Vaccinium vitis-idaea*)	37.6–251.1	20.1–48.5	n.d.	16.2–221.7	n.d.-47.5	n.d.	[106,107]
Gooseberry (*Ribes uva-crispa*)	43.0–49.0	1.67–3.53	n.d.	6.0–6.4	n.d.	n.d.	[106]
Black currant (*Ribes nigrum*)	31.66–31.72	n.d	21.30–21.32	17.47–17.49	n.d.	3.14–3.16	[105]
Red currant (*Ribes xpallidum*)	8.24–8.28	12.73–12.79	n.d.	n.d.	n.d	n.d.	[105]
Red raspberry (*Rubus idaeus*)	1.0–18.0	3.22–10.8	n.d.	0.6–9.4	210.0–220.0	n.d.-41.42	[105,106,108]
Strawberry (*Fragaria* × *ananassa*)	20.0–49.0	1.71–4.2	n.d.	n.d.-3.2	21.0–41.0	n.d.-68.4	[105,106,109,110]

n.d.: not detected.

**Table 9:** Example of flavonoid contents found in the most common red fruits.

Red Fruits	Flavonoids (mg/kg FW)				References
	Kaempferol	Myricetin	Quercetin	Luteolin	
Bilberry (*Vaccinium mytillus*)	n.d.	n.d.-21.0	n.d.-41.2	n.d.	[104,114]
Blueberry (*Vaccinium corymbosum*)	18.0	n.d.-34.0	31.0–83.0	n.d.-8.0	[104,115]
Cranberry (*Vaccinium oxycoccos*)	n.d.-6.1	43.0–230.0	73.0–250.0	n.d.	[114,115]
Lingonberry (*Vaccinium vitis-idaea*)	n.d.-10.3	n.d.	n.d.-34.7	n.d.	[114]
Gooseberry (*Ribes uva-crispa*)	n.d.-19.0	n.d.	n.d.-22.0	n.d.	[114]
Black currant (*Ribes nigrum*)	n.d.-23.0	n.d.-245.0	22.7–122.0	n.d.	[114]
Red currant (*Ribes xpallidum*)	n.d.-8.8	n.d.-42.9	n.d.-29.0	n.d.	[114,116]
Red raspberry (*Rubus idaeus*)	n.d.-1.0	n.d.	6.5–90.0	n.d.	[108,115]
Strawberry (*Fragaria* × *ananassa*)	n.d.-5.0	n.d.	6.0–19.0	n.d.	[108,115]
Crowberry (*Empetrum nigrum*)	n.d.	44.0–49.0	53.0–56.0	n.d.	[114]

n.d.: not detected.

**Table 10:** Example of anthocyanin contents found in the most common red fruits.

Red Fruits	Anthocyanin (mg/kg FW)						References
	Delphinidin	Cyanidin	Petunidin	Pelargonidin	Peonidin	Malvidin	
Bilberry (*Vaccinium mytillus*)	562.0–2913.0	488.0–955.0	437.0–705.0	n.d.	33.0–560.0	492.0–937.0	[123,124,125]
Blueberry (*Vaccinium corymbosum*)	405.0–768.0	82.8–379.0	294.8–319.0	n.d.	20.6–50.0	524.0–669.0	[110,123,125,126,127]
Cranberry (*Vaccinium oxycoccos*)	n.d.-10.8	13.2–313.0	n.d.-10.0	n.d.-185.3	n.d.-310.0	n.d.-25.0	[110,123,126,127]
Lingonberry (*Vaccinium vitis-idaea*)	n.d.	19–769.0	n.d.	n.d.	n.d.-6.0	n.d	[124,126]
Gooseberry (*Ribes uva-crispa*)	72.6–84.6	43.5–323.0	n.d.	n.d.	n.d.	n.d	[126,128]
Black currant (*Ribes nigrum*)	270.0–2940	166.5–1100.0	n.d.-2.0	n.d.	8.0–110	n.d.-180	[123,125,128]
Red currant (*Ribes xpallidum*)	n.d.	360.0–217.0	n.d.	n.d.	n.d.	n.d.	[123,126]
Red raspberry (*Rubus idaeus*)	n.d.	385.0–980.0	n.d.	9.0–660.0	n.d.	n.d.-44.9	[110,123,126]
Strawberry (*Fragaria* × *ananassa*)	n.d.	10.0–66.0	n.d.	162.0–336.4	n.d.	n.d.-8.5	[110,123,126,129]
Crowberry (*Empetrum nigrum*)	430.0–1183.0	550.0–775.0	240.0–421.0	n.d.	220–1037.0	997.0–1550.0	[123,126]

n.d.: not detected.

**Table 11:** Some examples of antioxidant activity values of red fruits by ORAC method.

Red Fruits	Antioxidant Activity	References
	ORAC (µmol Trolox/g FW)	
Bilberry (*Vaccinium myrtillus*)	14.4–122.7	[90,208]
Blueberry (*Vaccinium corymbosum*)	10.3–51.9	[108,209,210]
Cranberry (*Vaccinium oxycoccos*)	18.5–96.8	[211,212,213]
Lingonberry (*Vaccinium vitis-idaea*)	38.1	[211]
Gooseberry (*Ribes uva-crispa*)	17.0–41.5	[214]
Black currant (*Ribes nigrum*)	36.9–93.1	[214]
Red currant (*Ribes* *xpallidum*)	1.27–32.6	[213]
Red raspberry (*Rubus idaeus*)	7.8–45.2	[108,214,215]
Strawberry (*Fragaria* × *ananassa*)	20.2–22.1	[108,216]

## Data Availability

Data is contained within the article.
